# Novel Polymer-Based Smart Hydrogels: Design, Properties and Applications

**DOI:** 10.3390/gels12090784

**Published:** 2026-09-01

**Authors:** Ning Ma, Xue-Qing Zhan, Fang-Chang Tsai

**Affiliations:** Hubei Key Laboratory of Polymer Materials, Key Laboratory for the Green Preparation and Application of Functional Materials (Ministry of Education), Hubei Collaborative Innovation Center for Advanced Organic Chemical Materials, School of Materials Science and Engineering, Faculty of Life Sciences, Hubei University, Wuhan 430062, China

## 1. Introduction

Polymer-based smart hydrogels represent a rapidly evolving and highly interdisciplinary frontier in modern materials science. These three-dimensional cross-linked networks can absorb and retain substantial amounts of water or biological fluids while exhibiting a range of advanced functionalities, including stimuli responsiveness, self-healing, adhesion, and electrical conductivity, which are difficult to achieve with conventional rigid materials [1,2]. Their exceptional versatility, arising from tunable mechanical properties, favorable biocompatibility and biodegradability, and the capacity to respond to external stimuli such as pH, temperature, light, ions, and electric fields, has enabled their broad application across diverse fields. Consequently, smart hydrogels have attracted increasing attention in biomedicine, wearable electronics, energy storage, environmental remediation, and information security [3,4,5,6].

Recent advances in polymer chemistry and materials engineering have greatly expanded the design space and functional capabilities of smart hydrogels. Emerging fabrication strategies, including dynamic covalent crosslinking, enzymatic gelation, bioinspired self-assembly, and emulsion-based processing, have enabled increasingly precise control over hydrogel structures and properties [7,8]. Meanwhile, the incorporation of functional components such as MXenes, metal–organic frameworks, and conductive polymers has further broadened their electrical, optical, and mechanical properties, as well as responsive characteristics, facilitating applications in smart sensing, anti-counterfeiting, and targeted drug delivery [9,10]. Moreover, growing emphasis on sustainability is promoting the development of biodegradable materials, mild crosslinking approaches, and greener fabrication routes, reflecting a broader shift toward high-performance hydrogels with reduced environmental impacts [11].

The applications of smart hydrogels are expanding rapidly across a wide range of disciplines. In biomedicine, hydrogels have been explored as injectable self-healing wound dressings, localized drug delivery platforms, and biomimetic scaffolds for tissue regeneration [12]. In wearable technology, conductive hydrogel-based sensors enable sensitive and conformable monitoring of human motion and physiological signals [13]. In the energy sector, hydrogel-based systems have shown potential as functional media for gas storage and separation [14]. In construction and environmental engineering, hydrogel-functionalized materials offer solutions for soil stabilization, water purification, and sustainable building [15]. The convergence of these diverse application domains underscores the inherently interdisciplinary nature of hydrogel research and the need for platforms that facilitate knowledge exchange across chemistry, biology, engineering, and clinical science.

Building on the success of our first Special Issue, “Novel Polymer Gels: Synthesis, Properties, and Applications,” which showcased seven contributions spanning petrochemical engineering, biomedicine, construction, and environmental science, this second edition continues to highlight recent advances in research in this rapidly evolving field. The present Special Issue, titled “Novel Polymer-Based Smart Hydrogels: Design, Properties and Applications,” comprises seven original contributions that collectively reflect the breadth and depth of contemporary hydrogel science. These contributions explore innovative synthesis approaches, characterize novel hydrogel properties, and demonstrate transformative applications in wearable sensing, drug delivery, antibacterial materials, energy storage, anti-counterfeiting, and precision hydrogel bead fabrication. In the following section, we provide an overview of each contribution, with particular emphasis on its research objectives, principal findings, and broader significance to the hydrogel research community.

## 2. Overview of the Papers Published in This Special Issue

### 2.1. Smart Hydrogels for Wearable Sensing and Biomedical Applications

Multifunctionality and dynamic responsiveness represent important themes of this Special Issue. Wang et al. report the bio-inspired synthesis of an injectable, self-healing conductive hydrogel (PAA-Zn-silk Fibroin-MXene, denoted PZS-MXene) designed for wearable capacitive strain sensing. The hydrogel was constructed through Zn^2+^-mediated coordination crosslinking of polyacrylic acid (PAA), with silk fibroin forming a secondary network and conductive Ti_3_C_2_T_x_ MXene nanosheets incorporated to enhance electrical performance and network stability. The resulting hydrogel exhibited an elongation at break of 1750%, rapid self-healing within 30 s, and an electrical conductivity of 0.16 S/m. When assembled into a capacitive strain sensor, the PZS-MXene hydrogel operated over a wide strain range of up to 200%, with a maximum gauge factor of 1.78 in the 75–150% strain range, a fast response time of 0.2 s, and excellent cycling stability. As a wearable device, it accurately monitored the motion of finger, wrist, and elbow joints in real time. This work demonstrates the potential of combining dynamic ionic interactions, natural biopolymers, and two-dimensional conductive nanomaterials to develop flexible, self-healing, and sensitive hydrogel-based wearable sensors.

In a complementary biomedical study, Wang et al. present multi-responsive and antibacterial dynamic covalent hydrogels cross-linked by amphiphilic copolymer micelles. Several amphiphilic quaternized copolymers (Q-C8PEG-n) with tunable degrees of quaternization were synthesized and characterized for their thermoresponsive self-assembly behavior. Q-C8PEG-3 micelles containing diethanolamine units were cross-linked with phenylboronic acid-grafted hyaluronic acid (HA-PBA) through reversible boronic ester bonds stabilized by intramolecular B–N coordination. The resulting hydrogel exhibited pronounced responsiveness to pH, glucose and H_2_O_2_ environments; a porous structure with pore sizes of 8–15 μm; and biphasic Nile red release reaching approximately 52% after 24 h at pH 7.4. It also showed strong antibacterial activity against *Staphylococcus aureus*, with an inhibition zone of approximately 14 mm. This micelle-cross-linked dynamic covalent platform integrates stimuli responsiveness, controlled release, and antibacterial functionality, highlighting its potential for wound dressings and drug delivery systems.

Addressing a distinct biomedical challenge, Sipos et al. investigate gellan gum-based in situ hydrogels for the nasal delivery of risperidone-loaded polymeric micelles. Nasal drug delivery is hampered by rapid mucociliary clearance that limits drug residence time in the nasal cavity. To address this limitation, the authors combined gellan gum with different cellulose derivatives, including hydroxypropyl methylcellulose (HPMC), hydroxyethyl cellulose (HEC), and methylcellulose (MC), to construct ion-responsive matrices capable of gelation under simulated nasal conditions. The resulting formulations showed composition-dependent rheological and mucoadhesive properties, with several high-polymer-content systems exhibiting water-holding capacities above 80%. Incorporating the micelles into the hydrogel matrices reduced burst release and slowed short-term drug permeation, while enhanced mucoadhesion was expected to prolong nasal residence time. This work illustrates the potential of combining in situ gelling matrices with polymeric micelles for sustained nasal drug delivery.

### 2.2. Mechanically Robust and Stimuli-Responsive Hydrogels

A recurring challenge in hydrogel research is achieving a balance between mechanical robustness and multifunctional responsiveness. Ding et al. address this challenge through the development of multifunctional polyurethane hydrogels (PUGs) that integrate dynamic disulfide bonds and pendant tertiary amine groups into poly(ethylene glycol)-based networks via a solvent-exchange method. Structural characterization confirmed the formation of a stable cross-linked network with homogeneous porous morphology. Among the prepared hydrogels, PUG–II exhibited a tensile strength of 448 kPa, an elongation at break of 489%, and a compressive strength of 371 kPa at 90% strain, together with good fatigue resistance. The dynamic disulfide exchange mechanism enabled efficient room-temperature self-healing, with the PUGs achieving a healing efficiency of up to 94.5%. Meanwhile, reversible protonation of the tertiary amine groups imparted pronounced pH-responsive swelling behavior, with the equilibrium swelling ratio of PUG–I at pH 2.0 being 5.8 times higher than that at pH 12.0. This synergistic design provides an effective strategy for developing mechanically robust, self-healing, and pH-responsive polyurethane hydrogels for advanced smart-material applications.

### 2.3. Hydrogels for Energy Storage and Environmental Applications

Hua et al. explore an innovative application of hydrogels in methane storage by developing a hybrid reaction medium for hydrate-based methane storage. Biodegradable poly(vinyl alcohol-*co*-acrylic acid) (PVA-*co*-PAA) hydrogels were combined with dilute sodium p-styrenesulfonate (SS) solutions to synergistically promote methane hydrate formation. While the hydrogels or dilute SS solutions alone showed weak or negligible promoting effects, the hybrid media containing 1–4 mmol L^−1^ SS achieved methane storage capacities of 120.6–122.6 *v*/*v*. The highest storage capacity of 122.6 ± 1.9 *v*/*v* was obtained with 3 mmol L^−1^ SS at a hydrogel swelling ratio of 120 g g^−1^. The large surface area of the hydrogel particles provided abundant nucleation sites and an enlarged gas–liquid interface, while the dilute SS solution enhanced mass transfer through a capillary effect. Morphological observations further demonstrated that the hybrid medium mitigated wall-climbing hydrate growth and improved hydrate compactness compared with the bulk SS solution. Crucially, the system maintained stable promotion performance over at least 10 formation–dissociation cycles. This work highlights the potential of biodegradable hydrogel–SS hybrid systems as eco-friendly packing media for natural hydrate storage tanks, contributing to the development of sustainable methane storage technologies.

### 2.4. Advanced Hydrogel Platforms for Anti-Counterfeiting and Information Security

Yu et al. report a temperature–ultraviolet-responsive fluorescent hydrogel for intelligent anti-counterfeiting and information storage that addresses the limitations of traditional anti-counterfeiting materials, including low responsiveness, easy replication, and poor environmental stability. The authors designed a composite hydrogel (PAM/LMA/ZIF-8@Flu) in which fluorescein (Flu) was first confined within the pores of a zeolitic imidazolate framework (ZIF-8) via a one-pot method and then incorporated into a polyacrylamide/lauryl methacrylate (PAM/LMA) copolymer network. The hydrogel exhibited intense green luminescence under 365 nm UV illumination, and the fluorescence could be selectively quenched by Fe^3+^ and subsequently restored by PO_4_^3−^, enabling rewritable information storage. Additionally, the incorporation of sodium dodecyl sulfate (SDS) and sodium chloride (NaCl) endowed the hydrogel with a reversible temperature-dependent transparency transition, allowing concealed information to be revealed upon heating. By integrating ion-responsive fluorescence switching, thermally regulated optical transparency, and UV-assisted information readout, the hydrogel enables multi-level encryption and decryption. This work provides a promising strategy for developing rewritable, eco-friendly, and high-security intelligent anti-counterfeiting materials.

### 2.5. Precision Fabrication of Hydrogel Beads via Enzymatic Crosslinking

Liu and Zhang address the challenge of precisely controlling the size and size distribution of hydrogel beads during fabrication. The authors employed phenolic hydroxyl-functionalized poly(α,β-[N-(2-hydroxyethyl)-D,L-aspartamide]) (PHEA-HP), an enzymatically cross-linkable poly(aspartamide) derivative, to prepare hydrogel beads via an emulsion–enzymatic gelation process. Following single-factor experiments, a Box–Behnken design combined with a response surface methodology (BBD–RSM) was used to optimize three key emulsification parameters: the oil-to-water ratio, homogenization rate, and Span 80 dosage. The size distribution span was found to be more sensitive to these processing variables than bead size and was well described by a quadratic model, with R^2^ and adjusted R^2^ values above 0.99. The validated optimal conditions (an oil-to-water ratio of 10.3, a homogenization rate of 2930 rpm, and a Span 80 dosage of 2.0%) yielded spherical hydrogel beads with an average size of 14.0 ± 0.2 μm and a narrow size distribution span of 0.185 ± 0.010. This study provides a statistically guided approach for controlling the size distribution of enzymatically cross-linked hydrogel beads and offers useful processing guidance for their preparation in applications where bead uniformity is important.

## 3. Conclusions and Outlook

The seven contributions presented in this Special Issue collectively illustrate the remarkable versatility and rapidly expanding scope of polymer-based smart hydrogel research. Several overarching themes emerge from these studies and merit emphasis.

First, the integration of multiple functionalities within a single hydrogel platform represents a defining trend in the field. Whether through the combination of self-healing, conductivity, and stretchability in wearable sensor hydrogels; the convergence of multi-responsiveness and antibacterial activity in dynamic covalent networks; or the synergistic integration of fluorescence switching, thermal modulation, and UV-assisted fluorescence readout in anti-counterfeiting systems, researchers are increasingly designing hydrogels that transcend single-function limitations. This multi-functional paradigm reflects the growing recognition that real-world applications demand materials capable of simultaneous, coordinated responses to complex environmental stimuli.

Second, the studies in this issue highlight the critical importance of rational molecular and nanoscale design in achieving the desired hydrogel properties. The choice of crosslinking chemistry—whether ionic coordination, dynamic boronic ester bonds, enzymatic gelation, or disulfide exchange—fundamentally determines the mechanical, responsive, and self-healing characteristics of the resulting material. The strategic incorporation of functional nanomaterials such as MXene nanosheets and metal–organic frameworks further illustrates how hybrid architectures can endow hydrogels with capabilities far beyond those of their individual components. These advances underscore the need for a deep understanding of structure–property relationships to guide the rational design of next-generation hydrogel systems.

Third, sustainability and environmental compatibility are emerging as essential design criteria alongside performance metrics. The use of biodegradable PVA-co-PAA hydrogels for methane storage, enzymatic crosslinking strategies for poly(aspartamide)-based hydrogel beads, and the development of solvent-free, degradable anti-counterfeiting hydrogels all reflect a growing commitment to green chemistry principles within the hydrogel research community. As these materials transition from laboratory demonstrations to real-world deployment—whether in energy storage infrastructure, clinical drug delivery, or consumer anti-counterfeiting products—their environmental footprint and long-term biosafety will become increasingly important considerations.

Fourth, several contributions in this issue address the translation of hydrogel innovations from fundamental research toward practical applications. The recyclable hydrogel–SS hybrid medium for methane storage, the statistically optimized fabrication protocol for uniform hydrogel beads, and the mucoadhesive nasal delivery systems with controlled drug release and enhanced mucosal retention all exemplify the kind of engineering-oriented research needed to bridge the gap between laboratory discovery and industrial or clinical implementation. Future efforts in these areas will benefit from continued collaboration between materials scientists, process engineers, and end-users to ensure that hydrogel designs meet the practical requirements of scale, cost, regulatory compliance, and user acceptance.

Looking ahead, the field of polymer-based smart hydrogels is poised for transformative growth. Emerging directions include the development of 4D-printed hydrogel architectures that change shape and function over time, the integration of artificial intelligence and machine learning for accelerated hydrogel design and optimization, and the creation of biohybrid hydrogels that incorporate living cells or biological circuits for autonomous sensing and actuation. In biomedicine, the next generation of hydrogel-based therapeutics will likely feature patient-specific, on-demand drug release systems with closed-loop feedback mechanisms. In wearable technology, hydrogel sensors will evolve toward fully integrated, wireless, and self-powered systems capable of continuous multi-modal health monitoring. In sustainability-driven applications, hydrogels will play an increasingly important role in carbon capture, water remediation, and circular economy initiatives.

In conclusion, the studies gathered in this Special Issue demonstrate the increasing sophistication, interdisciplinarity, and application diversity of polymer-based smart hydrogel research. By pushing the boundaries of hydrogel design, synthesis, and functionality, these contributions collectively advance our ability to create materials that are smarter, more responsive, more sustainable, and more impactful than ever before. We anticipate that the concepts and findings reported herein will inspire further innovation, foster new cross-disciplinary collaborations, and accelerate the translation of hydrogel technologies into solutions that address some of the most pressing challenges in healthcare, energy, the environment, and information security.

## References

[1] Osada Y., Gong J.P. (1998). Soft and Wet Materials: Polymer Gels. Adv. Mater..

[2] Koetting M.C., Peters J.T., Steichen S.D., Peppas N.A. (2015). Stimulus-Responsive Hydrogels: Theory, Modern Advances, and Applications. Mater. Sci. Eng. R Rep..

[3] Rahmani P., Shojaei A., Sahabi M., Akbarizadeh M., Mahmoodi M., Zarghanishiraz A. (2025). A State-of-the-Art Review of Multi-Cross-Linked Hydrophobic Associated Hydrogels for Soft Electronic, Biomedical, and Environmental Applications. J. Mater. Chem. B.

[4] Tamate R. (2025). Development of Functional Polymer Gel Electrolytes and Their Application in Next-Generation Lithium Secondary Batteries. Polym. J..

[5] Wei J., Xiao P., Chen T. (2023). Water-Resistant Conductive Gels toward Underwater Wearable Sensing. Adv. Mater..

[6] Jiang H., Cheng Y., Zhang X., Li M., Wang Q., Yang L., Shuai C. (2025). Progress of Ionogels in Flexible Pressure Sensors: A Mini-Review. Polymers.

[7] Parvin N., Kumar V., Joo S.W., Mandal T.K. (2024). Cutting-Edge Hydrogel Technologies in Tissue Engineering and Biosensing: An Updated Review. Materials.

[8] Correa S., Grosskopf A.K., Lopez Hernandez H., Chan D., Yu A.C., Stapleton L.M., Appel E.A. (2021). Translational Applications of Hydrogels. Chem. Rev..

[9] He Q., Zhao C., Chen H., Wu T., Zeng C., Chen Y., Zhang C. (2024). Toward Next-Generation Wearable Sensors Based on MXene Hydrogels. J. Mater. Chem. A.

[10] Lim J.Y.C., Goh L., Otake K., Goh S.S., Loh X.J., Kitagawa S. (2023). Biomedically-Relevant Metal Organic Framework-Hydrogel Composites. Biomater. Sci..

[11] Babaei-Ghazvini A., Vafakish B., Patel R., Falua K.J., Dunlop M.J., Acharya B. (2024). Cellulose Nanocrystals in the Development of Biodegradable Materials: A Review. Int. J. Biol. Macromol..

[12] Liu J., Du C., Huang W., Lei Y. (2024). Injectable Smart Stimuli-Responsive Hydrogels: Pioneering Advancements in Biomedical Applications. Biomater. Sci..

[13] Liu D., Huyan C., Wang Z., Guo Z., Zhang X., Torun H., Mulvihill D., Xu B.B., Chen F. (2023). Conductive Polymer Based Hydrogels and Their Application in Wearable Sensors: A Review. Mater. Horiz..

[14] Li C., Tan K., Hua F., Wang C., Sun M., Wang F., Wang X. (2024). Eco-Friendly Hydrogels Serving as Water Carriers for Improving Methane Hydrate Formation and Dissociation Processes. Fuel.

[15] He Z., Shen A., Guo Y., Lyu Z., Li D., Qin X., Zhao M., Wang Z. (2019). Cement-Based Materials Modified with Superabsorbent Polymers: A Review. Constr. Build. Mater..

